# Efficient low-cost preparation of *trans*-cyclooctenes using a simplified flow setup for photoisomerization

**DOI:** 10.1007/s00706-016-1668-z

**Published:** 2016-02-22

**Authors:** Dennis Svatunek, Christoph Denk, Veronika Rosecker, Barbara Sohr, Christian Hametner, Günter Allmaier, Johannes Fröhlich, Hannes Mikula

**Affiliations:** Institute of Applied Synthetic Chemistry, TU Wien, Vienna, Austria; Institute of Chemical Technologies and Analytics, TU Wien, Vienna, Austria

**Keywords:** Photochemistry, Cycloadditions, Click chemistry, Alkenes, Isomerization, Bioorthogonal chemistry

## Abstract

**Abstract:**

Bioorthogonal ligations have emerged as highly versatile chemical tools for biomedical research. The exceptionally fast reaction between 1,2,4,5-tetrazines and *trans*-cyclooctenes (TCOs), also known as tetrazine ligation, is frequently used in this regard. Growing numbers of applications for the tetrazine ligation led to an increased demand for TCO compounds, whose commercial availability is still very limited. Reported photochemical procedures for the preparation of TCOs using flow chemistry are straightforward and high yielding but require expensive equipment. Within this contribution, we present the construction and characterization of a low-cost flow photoreactor assembled from readily accessible components. Syntheses of all commonly used *trans*-cyclooctene derivatives were successfully carried out using the described system. We are convinced that the presented system for photoisomerization will promote access to bioorthogonally reactive TCO derivatives.

**Graphical abstract:**

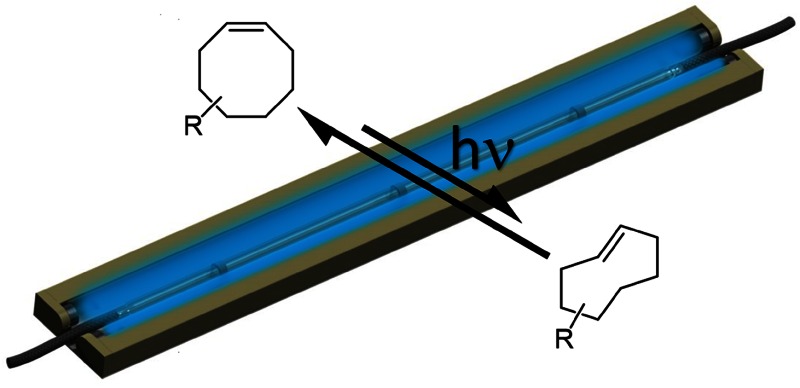

**Electronic supplementary material:**

The online version of this article (doi:10.1007/s00706-016-1668-z) contains supplementary material, which is available to authorized users.

## Introduction

From a chemists perspective biological systems are extraordinarily complex. Each individual cell combines a broad range of functional groups, macromolecules, enzymes, and organelles with different pH values as well as sub-compartments with oxidizing or reducing conditions. Stepping up from the single cell level to a higher organism adds another magnitude of complexity to the “in vivo chemical space”, which are far off simple mixtures usually present in a typical synthetic experiment.

Bioorthogonal chemistry, a term first introduced by Carolyn R. Bertozzi [1], is a tool that allows covalent bond formation within highly complex environments, using small molecule reaction partners. Those reactions (also referred to as “in vivo click chemistry”) meanwhile found numerous applications in fluorescent imaging, drug delivery, PET and SPECT imaging, radionuclide therapy, radiochemistry or drug target identification among several others [2–6]. High selectivity and fast ligation rates of the involved bioorthogonal groups are as important as the metabolic stability of reaction partners and ligation products, as well as their biocompatibility (also in regards of toxicity). A prominent example is the reaction of organic azides with cyclooctynes to form 1,2,3-triazoles, wherein the strained conformation of the cyclooctyne eliminates the need for (cytotoxic) copper(I)-catalysis thus also referred to as the copper-free click (CFC) or strain-promoted alkyne azide cycloaddition (SPAAC) [7]. SPAAC ligation rates are highly dependent on substitution motifs on the cyclooctyne moiety, whose synthesis is often cumbersome and low-yielding. Several improved and more easily accessible cyclooctyne derivatives have been reported, but reaction rates of SPAAC ligations are still limited to ~5 M^−1^ s^−1^. Nevertheless, besides many in vitro applications SPAAC has been successfully used for in vivo fluorescent imaging in developing zebrafish [8] as well as for pretargeting in positron emission tomography (PET) [9].

**Fig. 1:**
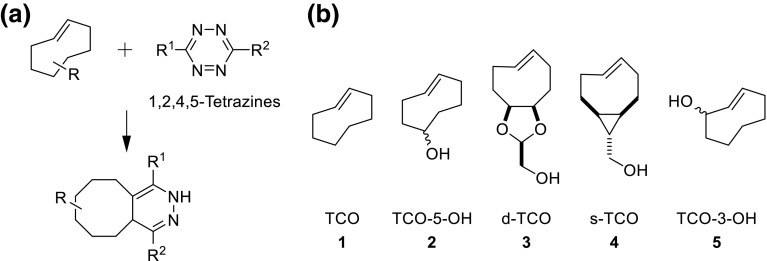
**a** Inverse electron demand Diels–Alder (IEDDA) reaction between *trans*-cyclooctenes and 1,2,4,5-tetrazines; **b** selected known *trans*-cyclooctene derivatives

A significant improvement was achieved by the groups of Joseph Fox [10] and Ralph Weissleder [11] in 2008, when they independently introduced the inverse electron demand Diels–Alder reaction (IEDDA) between 1,2,4,5-tetrazines (Tz) and strained dienophiles as highly efficient bioorthogonal reaction (Fig. 1a). When *trans*-cyclooctene (TCO) derivatives react with electron deficient Tz compounds second order rate constants of up to 3.3 × 10^6^ M^−1^ s^−1^ (25 °C, H_2_O) [12] were observed, making the IEDDA reaction several orders of magnitude faster than SPAAC and the fastest bioorthogonal ligation discovered so far. Hence, rapid ligation can be achieved even at very high dilutions frequently encountered within living systems. IEDDA has successfully been applied in pretargeted PET imaging [13, 14]. The recent development of tetrazines labeled with short-lived fluorine-18 [15–17] also allows imaging of long-circulating nanomedicines with short-lived isotopes as well as rapid radiolabeling of dienophile-tagged (bio)molecules [18–22], avoiding further purification steps. In pharmaceutical and biomedical research dienophile-tagged small molecules were used in vitro in combination with fluorogenic tetrazines [23–26] to visualize sites of drug accumulation at sub-cellular resolution. Tz ligations were also applied for target identification studies [27] and the synthesis of biocompatible polymers and hydrogels [28, 29].

Ligation rates of IEDDA conjugations are strongly dependent on steric and electronic influences on the tetrazine and the dienophile employed [30–32]. Norbornene and terminal alkenes were used as dienophiles, but low rates even with highly reactive Tz derivatives limit their use [33–35]. Cyclopropenes were introduced as dienophiles with less steric demand and higher reactivity compared to norbornene [36–38]. However, the most reactive dienophiles are *trans*-cyclooctenes (TCOs) (Fig. 1b). The parent compound *trans*-cyclooctene (TCO, **1**) is useful as reagent for ligation rate measurements using spectrophotometric techniques, but lacks a site for further derivatization thus making it unusable for bioconjugation. Consequently TCO derivatives including a functional group for further modification have been developed, out of which *trans*-cycloocten-5-ol (TCO-5-OH, **2**), which exists in two diastereomers, is the most frequently used. The diastereomer with axially configured hydroxyl group is formed in smaller amounts using photoisomerization and is thus often referred to as the “minor” TCO-5-OH (**2a**). **2a** shows moderately elevated reactivity compared to “major” (equatorial configuration) TCO-5-OH (**2e**). Nevertheless, steric hindrance makes further modifications of **2a** difficult, which is why **2e** is the most commonly used TCO in literature so far. TCO-5-OH has been proven to be reactive in vivo for prolonged times ranging to several days, but the mode of attachment and the microenvironment of the functionality is reported to influence the half-life inside the living system [39]. Annihilation of a cyclopropane ring to the cyclooctene core structure led to the development of (rel-1*R*,8*S*,9*R*,4*E*)-bicyclo[6.1.0]non-4-en-9-ylmethanol (s-TCO, **4**), which is one order of magnitude more reactive than TCO-5-OH. This dienophile was reported in 2011 by the Fox group [40], and is by far the fastest reacting TCO derivative known so far. Unfortunately high reactivity comes for the price of reduced stability, making s-TCO the choice whenever rapid reaction is more relevant than prolonged stability. Another highly reactive dienophile ((2*S*,3a*R*,9a*S*,*E*)-3a,4,5,8,9,9a-hexahydrocycloocta[*d*] [1, 3] dioxol-2-yl)methanol (d-TCO, **3**) [12], contains a dioxolane as structural motif enhancing its polarity and water solubility, which is a clear benefit when working in biological media. It exhibits IEDDA reactivity in between the rates for TCO-5-OH and s-TCO and is reported to display high stability. Another compound, *trans*-cycloocten-3-ol (TCO-3-OH, **5**), which also exists in an equatorial (**5e**) and axial configured (**5a**) isomer, deserves special attention due to its “click-to-release” behavior. The cytotoxic drug doxorubicin was conjugated to **5a** using a carbamate linkage on its 3-OH functionality leading to a pro-drug that can be activated by bioorthogonal click reaction with tetrazines [41]. This methodology gives rise to new therapeutic strategies, and the full potential of release chemistry is expected to unfold in years to come. The choice of which dienophile is suited best has to be made for every particular bioconjugation task depending on the necessities for ligation rate, stability, steric impact, and availability. Currently only the two isomers of TCO-5-OH, **2e** and **2a** are commercially available (**2e** also in activated form for conjugation reactions) and quite cost intensive when larger quantities are required.

**Fig. 2:**
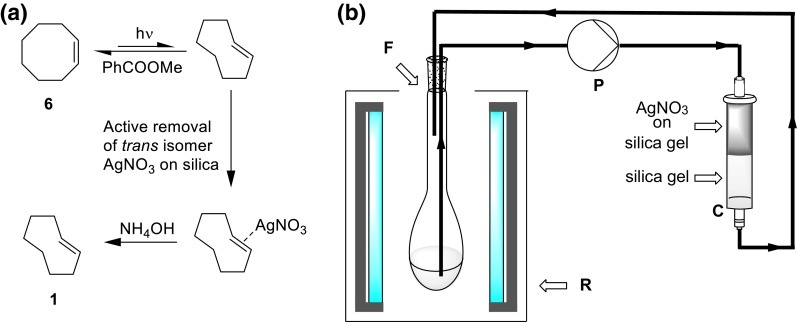
**a** Photochemical isomerization of *cis*-cyclooctene with active removal of *trans* compound; **b** Reaction setup used by Fox et al.: ***C*** column, ***P*** metering pump, ***F*** quartz glass flask filled with reaction solution, ***R*** Rayonet photochemical reactor

State of the art TCO synthesis was introduced by Fox and coworkers in 2008 and made these useful small molecules more accessible [42]. When *cis*-cyclooctenes are irradiated with UV-C (254 nm) in the presence of a singlet sensitizer an equilibrium between *cis*- and *trans*-isomers is reached (photoisomerization) [43, 44]. The *trans*-isomers, in contrast to the *cis*-compounds, form stable complexes with silver cations. Hence, passing the reaction mixture through a cartridge filled with AgNO_3_ on silica (AgNO_3_/SiO_2_) actively removes the TCO species from the equilibrium, allowing excellent reaction yields of TCOs from the respective *cis*-isomers in multigram quantities (Fig. 2a). Despite being broadly applicable for TCO synthesis the described setup uses a commercial UV reactor in which a quartz flask containing the reaction mixture has to be immersed (Fig. 2b). Both UV source and special glassware are cost intensive, and differently sized quartz flasks are necessary to maintain high flexibility regarding the scale of the reaction. Systems including flow photoreactors would eliminate this problem. Such reactors are commercially available (e.g. UV-150 photochemical reactor, Vapourtec Ltd.; Advanced-Flow™ G1 Photo Reactor, Corning^®^), but cost intensive and often require even more expensive flow chemistry equipment. We, therefore, aimed to develop and characterize an effective, versatile, and low-cost flow photoreactor using readily available components for the preparation of various TCO derivatives.

## Results and discussion

**Fig. 3:**
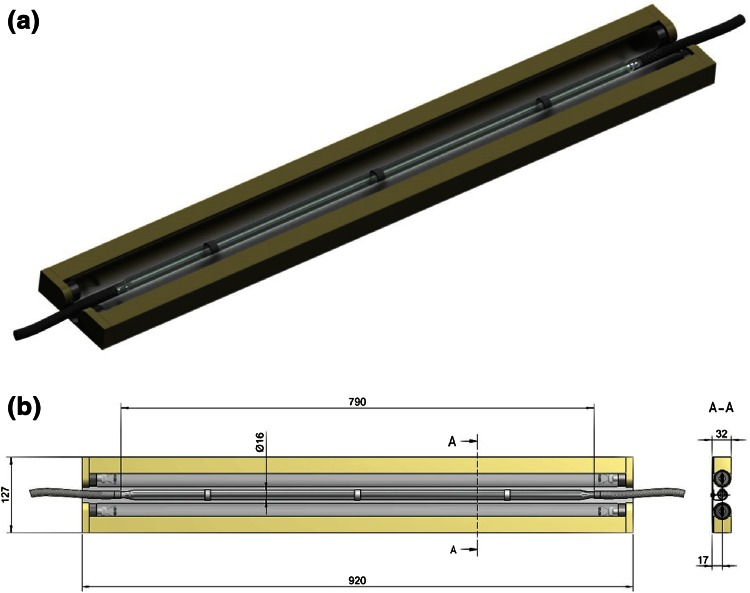
**a** 3D view of the flow photoreactor; **b** dimensions in mm

The most promising design featured a quartz glass tube (Ø 16 mm, length 790 mm) connected to tubes at both ends and a total volume of 165 cm^3^ as a flow cell. Two 55 W low-pressure mercury lamps (OSRAM, HNS 55 W OFR, T8, 90 cm) with a dominant wavelength of 254 nm were used for irradiation and placed alongside the quartz glass tube (Fig. 3). For a picture of the used setup see Electronic Supplementary Material (Fig. S1). This setup was mounted onto a back plate, covered with aluminum foil to prevent harmful radiation from exiting the system and aligned in vertical orientation. Solutions were pumped through the reactor upwards to allow for better filling of the reactor (see Electronic Supplementary Material). The total cost of this device was below 300€ while the costs for commercially available systems (photoreactor including lamps and quartz glass flasks) are significantly higher (2000–5000€ or even more).

**Fig. 4:**
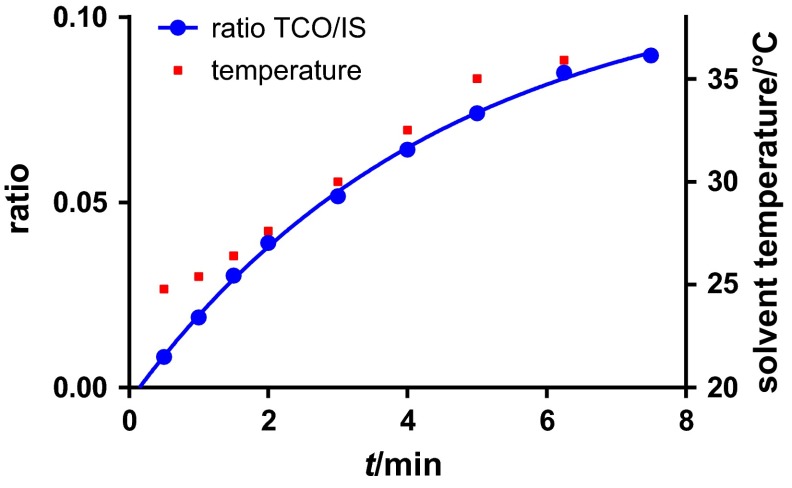
Development of TCO/IS ratio and temperature during isomerization

To determine the required time for reaching the equilibrium during photoisomerization a solution of *cis*-cyclooctene (**6**), methyl benzoate as singlet sensitizer and undecane as internal standard (IS, used for GC-FID measurements) in diethyl ether/hexanes (1/1) was irradiated without flow. Samples were taken after different irradiation times and analyzed by GC-FID. The temperature in the system reached 36 °C after about 6.5 min, which resulted in boiling of the solvent. The TCO/IS ratio was calculated over time and the data was fitted to a one phase association equation resulting in a predicted half-time of around 3.1 min (Fig. 4). The exact irradiation time for reaching the equilibrium could not be determined due to the rising temperature.

A flow rate of 100 cm^3^/min was chosen for photoisomerization, which results in a rather short dwell time of 99 s. This is beneficial since the rate of isomerization is highest within the first minutes and the increase of temperature is only moderate.

**Fig. 5:**
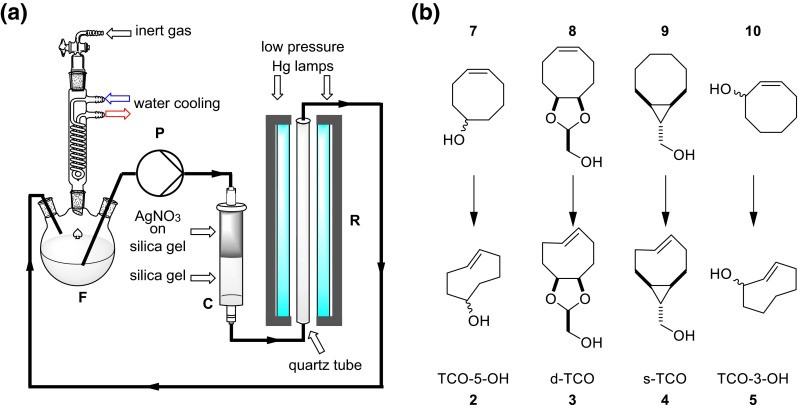
**a** Flow photoisomerization setup; **b** isomerization of compounds **2**, **3**, **4**, and **5**

For flow photoisomerization, the setup shown in Fig. 5a was used, for a picture see Electronic Supplementary Material (Fig. S2). An HPLC pump (**P**) was used to pass the reaction mixture from a reservoir flask (**F**) through a column (**C**) filled with silica and topped with AgNO_3_-impregnated silica (AgNO_3_/SiO_2_) and covered with aluminum foil, then through the vertically aligned flow reactor (**R**) back to the reservoir. The column was placed right after the pump and prior to the photoreactor to prevent damage due to backpressure. The use of the reservoir flask enables simple scaling of the reaction without the use of expensive quartz glass flasks in different dimensions, since containers of any size and shape can be used for **F**. Implementation of cooling/heating of the solution as well as reactions under inert gas atmosphere can easily be realized in this setup. In this case a 3-neck round bottom flask equipped with a condenser was used, which was cooled and put under inert gas atmosphere.

**Fig. 6:**
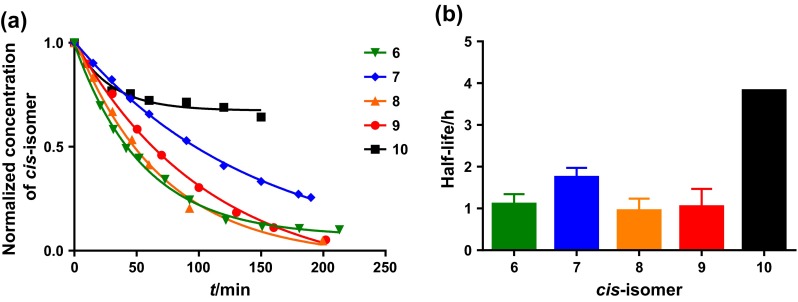
**a** Decline of CCO derivatives during photoisomerization; **b** half-lifes of CCO compounds during photoisomerization

This setup was used for the synthesis of *trans*-cyclooctene (**1**) as a test substance followed by isomerization of *cis*-cycloocten-5-ol (**7**), ((2*S*,3a*R*,9a*S*,*Z*)-3a,4,5,8,9,9a-hexahydrocycloocta[*d*] [1, 3] dioxol-2-yl)methanol (**8**) and (1*R*,8*S*,9*R*,4*Z*)-bicyclo[6.1.0]non-4-en-9-ylmethanol (**9**) as well as *cis*-cycloocten-3-ol (**10**) to their corresponding *trans*-isomers (Fig. 5b). Reactions were monitored by measuring the remaining *cis*-configured starting material by GC (Fig. 6a). Isomerizations of **6**, **7**, **8**, and **9** were performed at least in duplicates and revealed similar isomerization times and a mean half-life of 72 ± 24 min for all 9 reactions (Fig. 6b). The isomerization of **10** to TCO-3-OH (mixture of **5a/5e**) was significantly slower with a half-life of 3.8 h, which is in good agreement with the reported reaction time of 32 h [41] for this derivative in comparison to 6-26 h reported for other TCOs [12, 40, 42]. Furthermore, isolated yields were compared with previously reported results (Table 1).

**Table 1:** Comparison of yields for photoisomerization of **2**, **3**, **4**, and **5** reported in literature and those reached in this work

TCO	**2** (**2e**/**2a**)	**3**	**4**	**5** (**5e**/**5a**)
Literature	71 % [42] (47/24)	59 % [12]	74 % [40]	43 % [41] (25/18)
This work	47 % (32/15)	58 %	79 %	75 % (36/39)

## Conclusion

We were able to develop a low-cost and easy-to-build flow photoreactor that can be used for efficient synthesis of *trans*-cyclooctenes starting from respective *cis*-derivatives by photoisomerization. The setup was tested and characterized using different substituted and frequently used TCO derivatives. Isomerization was possible in relatively short time for all used substances with an average half-life of about 70 min with exception of **10** that showed a half-life of 3.8 h.

This simple design of a flow photoisomerization apparatus, which can easily be set up in any lab, enables the efficient and cheap production of TCO derivatives up to multigram scale.

## Experimental

Unless otherwise noted, all reagents were purchased from commercial suppliers and used without further purification. *Cis*-isomers **7** [42], **8** [12], **9** [40], and **10** [45] as well as AgNO_3_-impregnated silica gel (AgNO_3_/SiO_2_) [42] were prepared following known procedures. GC-FID measurements were performed on a Trace™ 1310 GC chromatograph from Thermo Scientific equipped with TR-5MS columns (length 15 m, I.D. 0.25 mm, film 1.0 µm). ^1^H and ^13^C NMR spectra were recorded on a Bruker AC 200 or Bruker Avance UltraShield 400 spectrometer at 20 °C.

### Kinetics of photoisomerization

The flow photoreactor as illustrated in Fig. 3 was closed at the bottom and filled with a 10 mM solution of 187 mg *cis*-cyclooctene (1.7 mmol), 231 mg methylbenzoate (1.7 mmol), and 265 mg *n*-undecane (1.7 mmol) in 170 cm^3^ hexanes/Et_2_O (1:1). A resistance thermometer was placed inside the tube to follow the temperature of the solvent. The lamps were turned on and samples were taken at different time points and analyzed by GC-FID.

### Photoisomerization and synthesis of TCO compounds: general procedure

The setup shown in Fig. 5a was used. The column was filled with silica gel, topped with AgNO_3_/SiO_2_ (10 %) and flushed with solvent. Then the reservoir was filled with solvent and put under argon atmosphere. The pump was turned on and set to a flow rate of 100 cm^3^/min. After the solvent had filled the whole system *cis*-cyclooctene (final concentration between 5 and 20 mM), methyl benzoate and, if applicable, *n*-undecane was slowly added to the reservoir over the course of several minutes to ensure homogeneous distribution of all reactants. Afterwards the solution was pumped through the system for another 20 min. The lamps were turned on and the reaction progress was monitored by GC-FID following the decline of the starting material in the reservoir flask using either methyl benzoate or *n*-undecane as internal standard. The reservoir flask was cooled with ice-water during isomerization. After the reaction was completed, as indicated by consumption of the starting material, the lamps were turned off. The column was flushed with generous amounts of the solvent as used during isomerization (>60 cm^3^/mmol of *cis*-configured starting material) and then dried with compressed air. Silica and AgNO_3_/SiO_2_ were taken out of the column, stirred in a mixture of concentrated NH_4_OH (37 %) and dichloromethane (each 10 cm^3^/g AgNO_3_/SiO_2_) and filtrated. The filter cake was washed twice with concentrated NH_4_OH (5 cm^3^/g AgNO_3_/SiO_2_) and dichloromethane (5 cm^3^/g AgNO_3_/SiO_2_). The organic layer was separated, washed twice with water, dried over Na_2_SO_4_, and concentrated. Column chromatography was used for further purification if needed.

#### *trans*-*Cyclooctene* (**1**)

Following the general procedure using 9.50 g **6** (86 mmol), 12.30 g methyl benzoate (91 mmol), 130 g AgNO_3_/SiO_2_ (10 %), 100 g SiO_2_, and 3500 cm^3^ Et_2_O/hexanes (1:99); 5.50 g (50 mmol, 58 %) of pure **1** were obtained without further purification. Spectroscopic data matched those reported in the literature [42].

#### *TCO*-*5*-*OH, rel*-*(1R,4E,pR)*-*cyclooct*-*4*-*enol* (**2e**) and *rel*-*(1R,4E,pS)*-*cyclooct*-*4*-*enol* (**2a**)

Following the general procedure using 2.03 g **7** (11.9 mmol), 2.00 g methyl benzoate (14.7 mmol), 27 g AgNO_3_/SiO_2_ (10 %), 18 g SiO_2_, 700 cm^3^ Et_2_O/hexanes (9:1), 1.00 g *n*-undecane (6.4 mmol); the crude product was purified by column chromatography (90 g silica, hexanes/ethyl acetate, 4:1) to yield 658 mg (32 %, 5.2 mmol) of equatorial product (**2e**) and 313 mg (15 %, 2.5 mmol) of axial product (**2a**). Spectroscopic data matched that reported in the literature [42].

#### *d*-*TCO, ((2* *s,3aR,9aS,E)*-*3a,4,5,8,9,9a*-*hexahydrocycloocta[d][*1, 3*]dioxol*-*2*-*yl)methanol* (**3**)

Following the general procedure using 1.50 g **8** (8.2 mmol), 2.00 g methyl benzoate (14.7 mmol), 19 g AgNO_3_/SiO_2_ (10 %), 20 g SiO_2_, 500 cm^3^ Et_2_O/hexanes (1:1), 1.00 g *n*-undecane (6.4 mmol); column chromatography (Et_2_O/hexanes, 2:1) was performed to yield 875 mg (58 %, 4.8 mmol) of desired product **3**. Spectroscopic data matched that reported in the literature [12].

#### *s*-*TCO, (rel*-*1R,8S,9R,4E)*-*bicyclo[6.1.0]non*-*4*-*en*-*9*-*ylmethanol* (**4**)

Following the general procedure using 1.50 g **9** (9.9 mmol), 1.30 g methyl benzoate (9.5 mmol), 33 g AgNO_3_/SiO_2_ (10 %), 50 g SiO_2_, 1000 cm^3^ Et_2_O/hexanes (1:1), 1.00 g *n*-undecane (6.4 mmol); 1.19 g (79 %, 7.8 mmol) of pure **4** were obtained without chromatographic purification. Spectroscopic data matched that reported in the literature [40].

#### *TCO*-*3*-*OH, (1RS,2RS)*-*trans*-*cyclooct*-*2*-*enol* (**5e**) and *(1SR,2RS)*-*trans*-*cyclooct*-*2*-*enol* (**5a**)

Following the general procedure using 2.00 g **10** (15.8 mmol), 2.15 g methyl benzoate (15.8 mmol), 35 g AgNO_3_/SiO_2_ (10 %), 40 g SiO_2_, 800 cm^3^ Et_2_O/hexanes (1:1), 2.50 g *n*-undecane (15.8 mmol); the crude product was purified by column chromatography (90 g silica gel, 10-50 % ethyl acetate in hexanes) to yield 694 mg (35 %, 5.5 mmol) of axial isomer **5a**, 669 mg (33 %, 5.3 mmol) of equatorial isomer **5e** and 146 mg of a mixture (**5a**/**5e** = 1.3:1, 7 %, 1.2 mmol). NMR spectra matched those reported in the literature [41].

## Electronic supplementary material

Supplementary material 1 (PDF 465 kb)
