# Supplementary material for: Efficient low-cost preparation of trans-cyclooctenes using a simplified flow setup for photoisomerization
Source: Monatsh Chem. 2016 Feb 22;147:579–85. doi: 10.1007/s00706-016-1668-z (PMC4785212; doi:10.1007/s00706-016-1668-z)

Supporting Information

Efficient low-cost preparation of *trans*-cyclooctenes using a simplified flow setup for photoisomerization

Dennis Svatunek[1, 2] ● Christoph Denk[1] ● Veronika Rosecker[1] ● Barbara Sohr[1] ● Christian Hametner[1] ● Günter Allmaier[2] ● Johannes Fröhlich[1] ● Hannes Mikula[1]

✉ Hannes Mikula

hannes.mikula@tuwien.ac.at

[1] Institute of Applied Synthetic Chemistry, TU Wien, Vienna, Austria

[2] Institute of Chemical Technologies and Analytics, TU Wien, Vienna, Austria

Flow reactor

Fig. S1 Flow photoreactor; 1) 55W OSRAM HNS 90 cm T8 OFR lamps; 2) Quartz tube

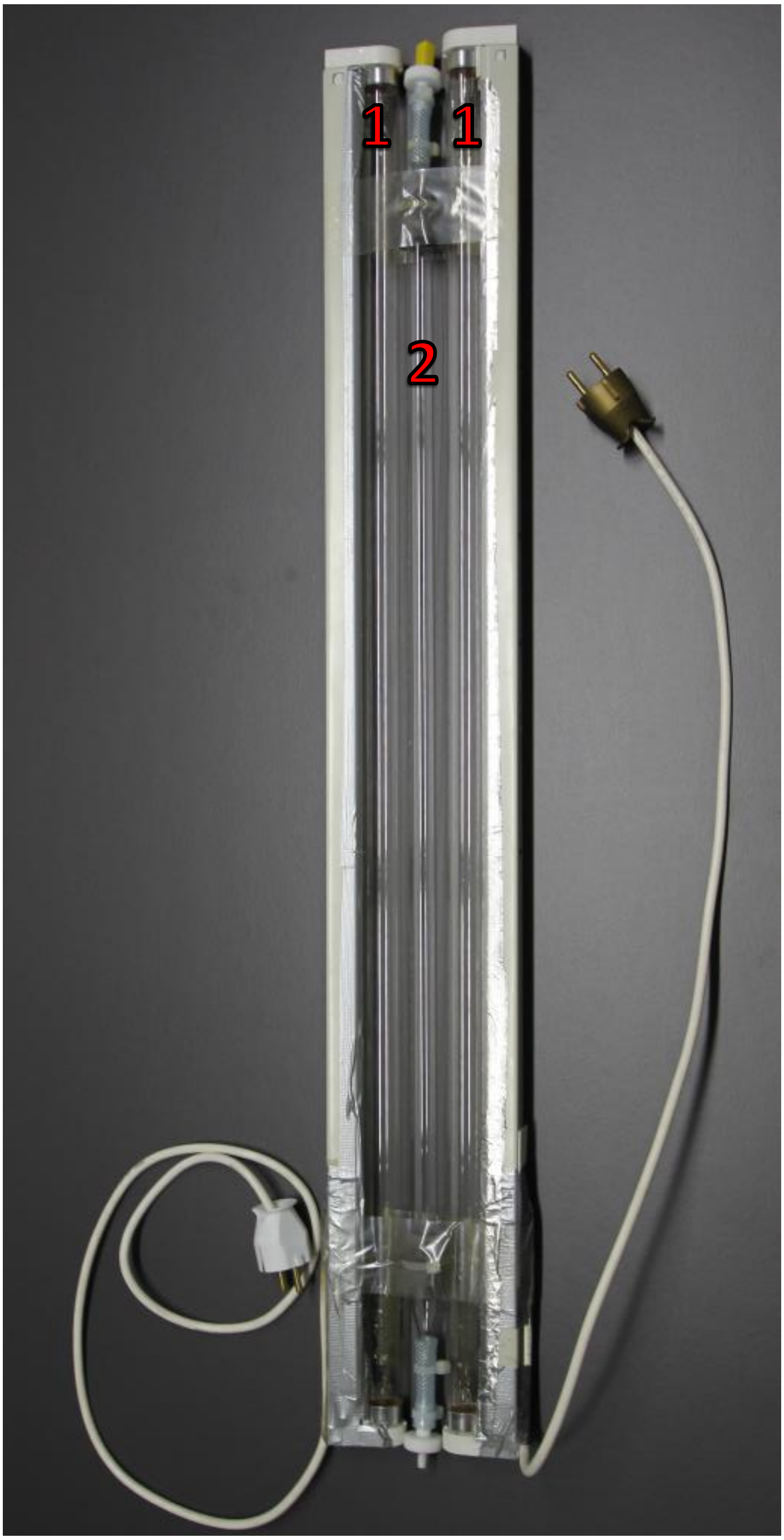

Photoisomerization setup

Fig. S2 Photoisomerization setup; **1**: Reservoir flask equipped with condenser and cooled with ice water; **2**) BESTA HPLC HD 2-2000 pump; **3**) Column filled with silica gel topped by $AgNO_3$ on silica gel (10%) ; **4**) Flow photo reactor

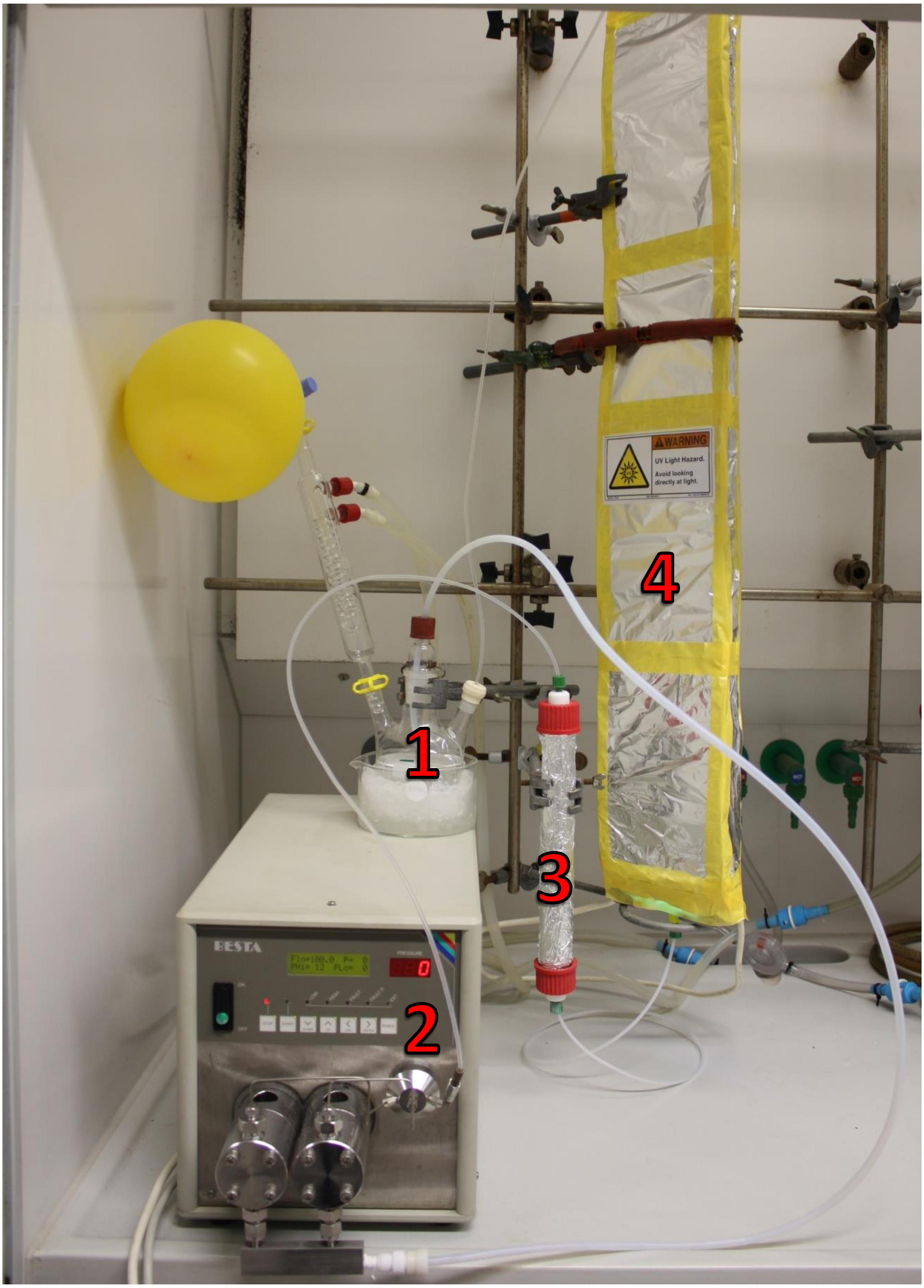

Supplement: Supplementary file 1 — Supplementary material 1 (PDF 465 kb) [file 706_2016_1668_MOESM1_ESM.pdf]
